# Topographic and quantitative correlation of structure and function using deep learning in subclinical biomarkers of intermediate age-related macular degeneration

**DOI:** 10.1038/s41598-024-72522-9

**Published:** 2024-11-15

**Authors:** Klaudia Birner, Gregor S. Reiter, Irene Steiner, Gábor Deák, Hamza Mohamed, Simon Schürer-Waldheim, Markus Gumpinger, Hrvoje Bogunović, Ursula Schmidt-Erfurth

**Affiliations:** 1 Laboratory for Ophthalmic Image Analysis (OPTIMA), Department of Ophthalmology and Optometry, Medical University of Vienna, Vienna, Austria; 2 Center for Medical Data Science, Institute of Medical Statistics, Medical University of Vienna, Vienna, Austria; 3 Department of Ophthalmology and Optometry, General Hospital of Vienna, Währinger Gürtel 18-20, 1090 Vienna, Austria

**Keywords:** Diseases, Eye diseases, Macular degeneration, Diagnostic markers

## Abstract

To examine the morphological impact of deep learning (DL)-quantified biomarkers on point-wise sensitivity (PWS) using microperimetry (MP) and optical coherence tomography (OCT) in intermediate AMD (iAMD). Patients with iAMD were examined by OCT (Spectralis). DL-based algorithms quantified ellipsoid zone (EZ)-thickness, hyperreflective foci (HRF) and drusen volume. Outer nuclear layer (ONL)-thickness and subretinal drusenoid deposits (SDD) were quantified by human experts. All patients completed four MP examinations using an identical custom 45 stimuli grid on MP-3 (NIDEK) and MAIA (CenterVue). MP stimuli were co-registered with corresponding OCT using image registration algorithms. Multivariable mixed-effect models were calculated. 3.600 PWS from 20 eyes of 20 patients were analyzed. Decreased EZ thickness, decreased ONL thickness, increased HRF and increased drusen volume had a significant negative effect on PWS (*all p* < 0.001) with significant interaction with eccentricity (*p* < 0.001). Mean PWS was 26.25 ± 3.43 dB on MP3 and 22.63 ± 3.69 dB on MAIA. Univariate analyses revealed a negative association of PWS and SDD (*p* < 0.001). Subclinical changes in EZ integrity, HRF and drusen volume are quantifiable structural biomarkers associated with reduced retinal function. Topographic co-registration between structure on OCT volumes and sensitivity in MP broadens the understanding of pathognomonic biomarkers with potential for evaluation of quantifiable functional endpoints.

## Introduction

Recent breakthroughs in the treatment of non-exudative age-related macular degeneration (AMD) induce an urgent need for structural and functional monitoring of conversion and progression in early and intermediate AMD (iAMD)^1,2^. In dry AMD, conventional visual acuity testing typically remains unimpaired despite disease progression with pathognomonic biomarkers manifest on optical coherence tomography (OCT) imaging and evidence of reduced global sensitivity (PWS) in microperimetry (MP)^3–5^.

Advanced analysis of OCT imaging revolutionizes the display of retinal morphology and allows for a fast and non-invasive visualization of retinal morphology for AMD detection and follow-up^6^. Particularly, novel developments in machine learning and deep learning (DL) provide clinicians with precise and objective algorithms for AMD biomarker localization and offer a reliable automated quantification of subclinical biomarkers based on OCT imaging, providing unprecedented insight into the pathomechanisms of AMD disease and improving patient management and screening^7,8^. Recent observations indicated that outer nuclear layer (ONL) thickness, ellipsoid zone (EZ) integrity loss such as EZ thickness reduction, drusen and hyperreflective foci (HRF) volumes can be automatically quantified in OCT scans and may be related to a distinct negative impact on topographic retinal sensitivity measured by MP in iAMD and advanced AMD^3,9–11^. Longitudinal studies showed that central drusen volume, HRF and subretinal drusenoid deposits (SDD) are high-risk indicators for subsequent progression to complete RPE atrophy^12^. Moreover, SDD are increasingly recognized as an independent biomarker strongly associated with an increasing risk of late AMD development with an early impact on retinal dysfunction^13–15^. However, despite engaged studies on the correlation between such morphological features and retinal function, little conclusive evidence has been provided to systematically quantify the structure/function correlation on a topographic level and superimpose such subclinical hallmarks with OCT B-scans^13,15^. Available studies largely focus on localized changes of specific biomarkers or mean sensitivity associated with overall biomarker presence and do not provide a full spectrum of high-risk biomarkers, also considering the dependency of sensitivity values on foveal eccentricity^3,14–17^.

With novel treatments for late non-exudative AMD and the search for early therapeutic targets, there is an emerging need for functional endpoints, as requested by regulatory agencies to address the shortcomings of best-corrected visual acuity (BCVA) testing^18^. Early and automated quantification of progression-relevant AMD biomarkers and a precise correlation with neurosensory function in MP offers essential insights into disease activity and progression. The goal of this prospective study was to topographically quantify structural changes in iAMD and assess the differential impact of the key biomarkers e.g. ONL thickness, EZ thickness, SDD, HRF and drusen volume on PWS in two different commercially widely available microperimetry devices under standardized settings.

## Methods

### Study population

Patients above 50 years of age were recruited at the Department of Ophthalmology and Optometry at the Medical University of Vienna, Austria. Participants were included after giving informed written consent following a study protocol complying to the tenets of the Declaration of Helsinki and approved by an independent ethics committee (Ethikkommission, Medizinische Universität Wien, Österreich, Nr. 1399/2021). Definition of early to intermediate AMD was based on Ferris et al. with a minimal drusen width of 65–125 µm with or without pigmentary abnormalities^19^. Exclusion criteria were any signs of late AMD including the presence of cRORA (defined as an RPE attenuation > 250 µm, choroidal hypertransmission > 250 µm and overlying ellipsoid zone degeneration)^20^ or macular neovascularisation (MNV)^21^, history of glaucoma or any visual field impairment (e.g. PION, AION), any significant media opacities, amblyopia and refractive errors above ± 5 dpt. Only one eye per patient was included. In cases where both eyes were eligible the one with better OCT imaging quality was selected.

### Microperimetry procedures

**Fig. 1:**
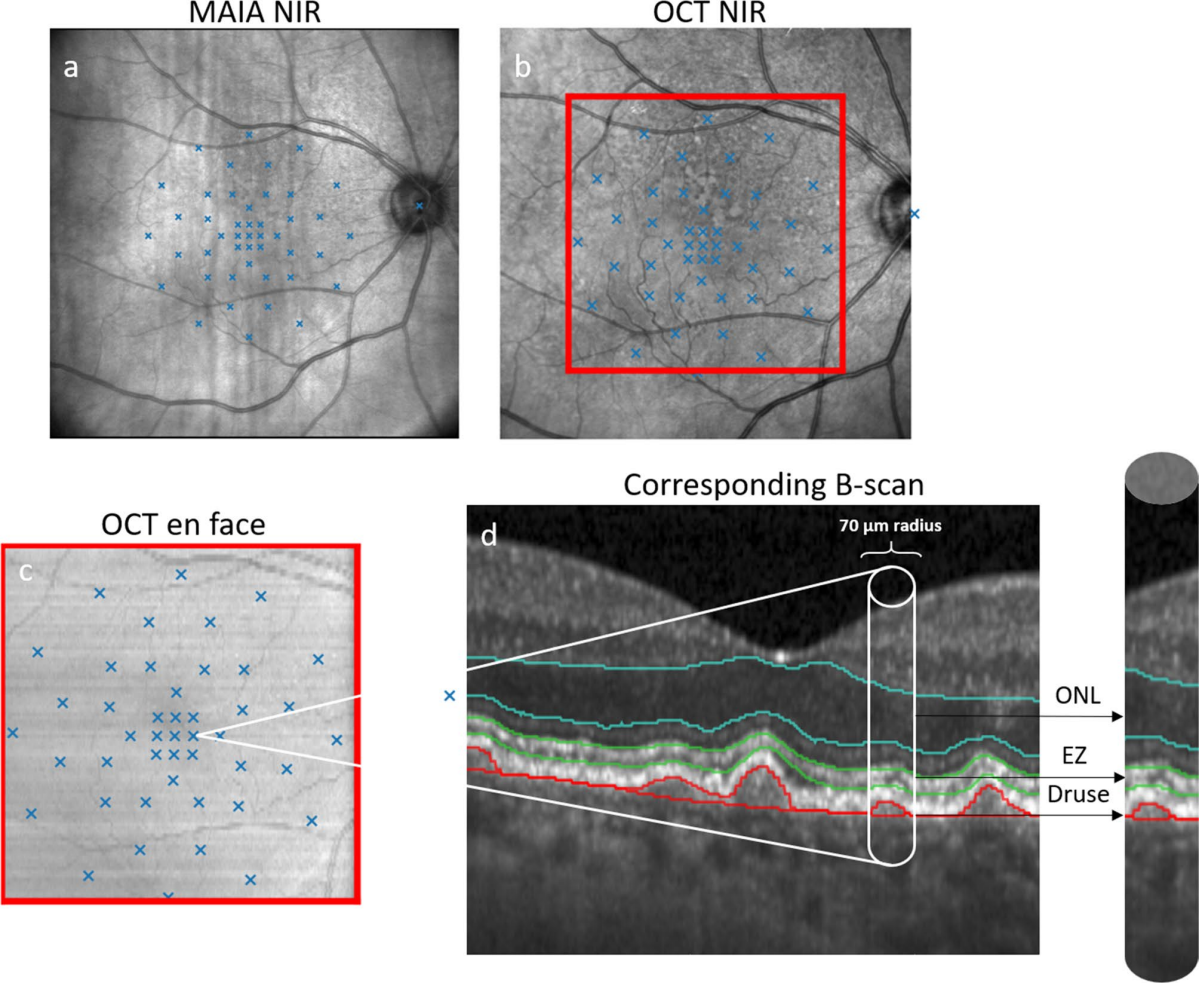
Example of multiple steps within the registration process representing the position of our in-house developed MP grid. From MAIA-NIR (**a**) to NIR (**b**) and the corresponding en face location of the MP points on OCT (**c**) from one of the study participants. The schematic representation of the corresponding B-scan and the area of biomarker quantification within the B-scan is displayed in (**d**). The biomarkers were quantified within a 70 µm radius around each stimulus point and vessel position were used as junctional points during the registration process. *NIR* near-infrared reflectance, *OCT* optical coherence tomography, *MP* microperimetry, *ONL* outer nuclear layer, *EZ* ellipsoid zone.

All eligible patients underwent four microperimetry tests during one visit after pupillary dilation with 0.5% Tropicamid. Two consecutive test runs were conducted with the Microperimetry MP-3 (NIDEK CO., Ltd., Gamagori, Japan) in the standard photopic setting (background 31.4. asb, 10 cd/m^2^) and two test runs on the Macular Integrity Assessment device (MAIA, CenterVue S.p.A. (iCare), Padova, Italy) in the standard mesopic setting (background 4 asb, 1.27 cd/m^2^). Therefore, each patient underwent four microperimetry tests in total divided into run 1 (meaning the first examination) and run 2 (meaning the second examination) per device. The testing conditions were chosen to mirror the settings of both devices in daily clinical practice and elaborate on both, photopic and mesopic conditions. Which device test was performed first was randomized to minimize any learning or weariness bias. The second consecutive examination was performed with the device-specific follow-up function after a mandatory 10-min break. The tests were performed under standardized settings in a dark and quiet room without windows (< 1 lx). The fellow eyes were covered with an eye patch. A 4–2 staircase stimulation strategy was selected, while the first stimulus was set at 17 dB for both devices and test runs. The light stimuli were set at 45 points around the fovea in a customized test grid with a stimulus size of Goldmann III for the duration of 200 ms (Figs. 1 and 3) PWS was tested in a range from 0 to 34 dB for MP-3 and from 0 to 36 dB for MAIA. In case where − 1 dB values were provided by the MAIA procedure, values were adjusted to 0 dB for consistency with the measurement range obtained from MP-3.

### Imaging procedures and image analysis

**Fig. 2:**
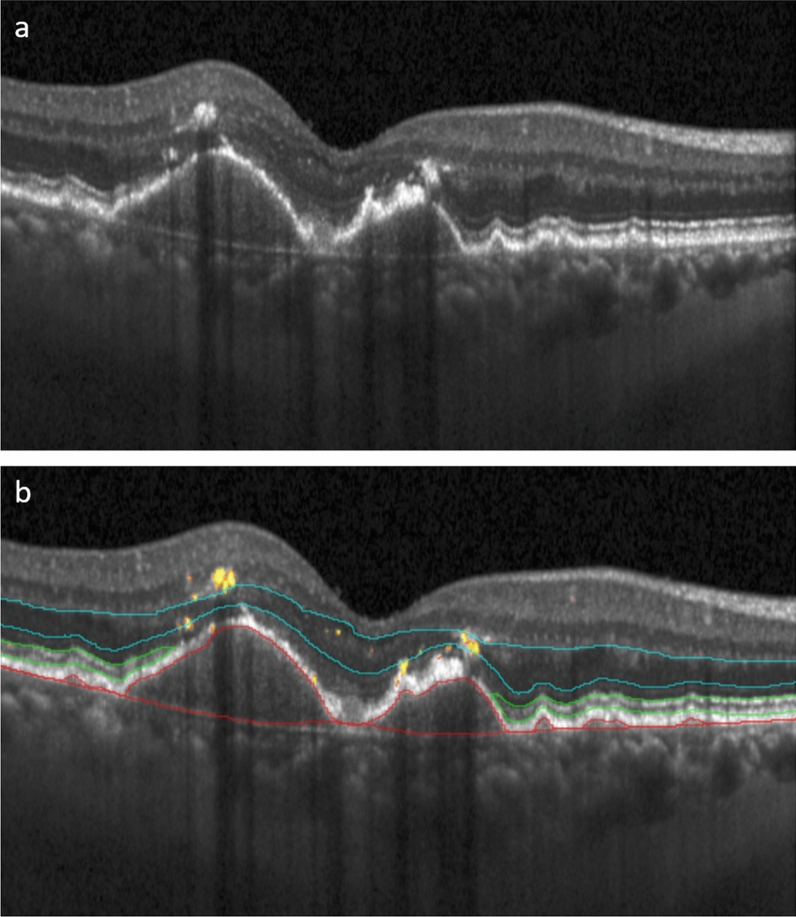
Example of automated biomarker quantification in intermediate AMD. ONL thickness measured between the blue lines (inner boundary of ELM to outer boundary of OPL), EZ thickness measured between the green lines (inner boundary of EZ to outer boundary of interdigitation zone), drusen volume (inner boundary of BM to outer boundary of RPE) and HRF volume as yellow “dots” (**b**). *ONL* outer nuclear layer, *EZ* ellipsoid zone, *OPL* outer plexiform layer, *ELM* external limiting membrane, *HRF* hyperreflective foci.

The diagnosis of iAMD was based on spectral-domain OCT performed with Heidelberg Spectralis HRA + OCT (Heidelberg Engineering, Heidelberg, Germany) using 97 B-scans in a 20 × 20° macular cube, centered on the fovea. EZ thickness was calculated based on automated segmentation of a previously validated deep-learning algorithm^22^.The EZ layer was defined from the outer boundary of interdigitation zone to the inner layer of the ellipsoid zone (EZ). The ONL was measured from the outer border of the outer plexiform layer (OPL) to the external limiting membrane (ELM). To achieve the highest precision of the ONL thickness measurements, if necessary, the DL-based segmentations of the outer border of the OPL were manually corrected and Henle fibre’s layer was included into the ONL thickness value^23^. ELM annotations were manually corrected when necessary. HRF and drusen volumes were derived from a fully automated segmentation by a convolutional neural network as previously described^24^. For HRF volume, a previously published threshold of 0.06 nl was implemented and only values above this threshold were considered as HRF feature^16^. The boundaries for drusen volume calculations were defined from Bruch’s membrane (BM) to the outer border of the retinal pigment epithelium (RPE) (Fig. 2)^16^.

### Subretinal drusenoid deposits

Manual pixel-wise annotations were performed using an in-house developed software for each of the 97 B-scans in all OCT volumes of all patients that presented with SDD. SDD were considered as present if at least one B-scan had a characteristic subretinal deposition with a typical SDD pattern in the corresponding near-infrared reflectance (NIR) image. SDD were annotated based on previous OCT definition as an accumulation of reflective material above the RPE to Bruch’s membrane band, impinging and/or penetrating the EZ^13^. All annotations were performed by trained expert readers. Questionable cases were discussed by the team of readers with the annotation leader of the Vienna Reading Center (G.D.) until a consensus was reached. An example of SDD annotations is presented in Fig. 6.

### Co-registration between MP and OCT feature topography

**Fig. 3:**
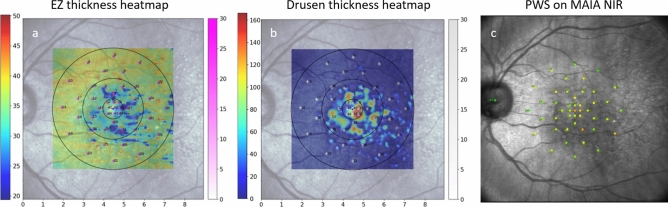
En face representation via thickness heatmaps (scale on the left) of quantified biomarkers of EZ thickness (**a**) and drusen thickness (**b**) from the OCT volume. The location of the in-house developed MP grid visible within the heatmap with its respective PWS scale (scale on the right). Numbers within the MP grid in a-b represent the number of the stimulus, not the sensitivity at the respective MP point. Figure (**c**) displays PWS of each stimulus as presented on the MAIA device. Stimuli points within drusen areas and lower EZ thickness have lower PWS (orange and yellow colours in **c**). *EZ* ellipsoid zone, *OCT* optical coherence tomography, *MP* microperimetry, *PWS* pointwise sensitivity.

Registration between both MP devices and the respective OCT feature and volume was performed using an in-house developed algorithm as previously published^16,25,26^. After data export from each respective device, pixel positions for each stimulus point obtained from the MAIA-NIR and the MP-3-color fundus photography (CFP) were established. Both the MP-3-CFP and the MAIA-NIR were co-registered with the NIR image that was acquired during the OCT volume scan procedure. The registration of the positions to the NIR image was derived from an automated customized algorithm performing retinal vessel segmentation in both images (MAIA-NIR/MP3-CFP and NIR) to define and connect the junctional points. To exclude any misalignment, the automated registration was furthermore reviewed by a human expert (S. SW). In case of deviation of the respective MP stimuli points between the MAIA-NIR/MP3-CFP and the NIR, manual correction was performed by marking the junctional points on both images and calculating the transformation matrix based on the “least square” error method. As the positions on NIR were already registered with each B-scan of the OCT volume, the MP stimuli could be accurately located on the corresponding B-scans (Figs. 1 and 3). OCT pixel spacing was used to determine all pixels representing each respective stimulus point within the 70 μm radius to quantify any biomarker, e.g., layer thickness at the precise location of each of the 45 stimuli.

### Statistical analyses

Statistical analyses were carried out using IBM SPSS and R 4.3.2^27^. Quantitative variables are summarized as mean ± standard deviation if approximately normally distributed and as median [minimum—maximum] otherwise. A multivariable mixed-effect model was used to examine the impact of independent variables device, run, age, eccentricity, ONL thickness, EZ thickness, drusen, HRF, and SDD volume on point-wise retinal sensitivity. Variable selection was performed using the R-function dredge (R-package MUMIn). The global model was a mixed model (function lme, R-package nlme^28^) fitted by maximum likelihood (ML) with patient as random factor and the fixed factors listed above, whereby also the interactions between EZ thickness and ONL thickness, SDD volume and drusen volume, as well as the interactions between eccentricity and drusen volume, SDD volume, EZ thickness and ONL thickness and the interaction between ONL thickness and drusen volume were considered. For model selection, the Bayesian Information Criterion (BIC)-criterion was used^29^. To perform variable selection using the function dredge of the R-package MuMIn, first a set of candidate models is fitted that include different combinations of predictor variables. The dredge function then systematically evaluates all possible models and calculates their BIC values. Afterwards, the model with the lowest BIC is identified as the best fitting model. Due to missing values with MP points located outside of the OCT area, 3.545 observations out of 3.600 observations were included in the model.

Independent variables, that were selected in the model with the smallest BIC were included in a mixed model with patient as random factor using REML estimation, whereby all valid observations were considered (3545 observations). The marginal coefficient of determination for generalized mixed-effect models *Marginal R*^2^_*GLMM*_ represents the variance explained by the fixed effects calculated with R-package MuMIn, R-function r.squaredGLMM^30^. The correlation between independent variables was analyzed with the Spearman correlation coefficient (*r*_*s*_). Due to the high proportion of values with value “0”, analyses were repeated with the independent variables drusen, SDD, and HRF volume dichotomized in absent/present. Since SDD volume was not included in the final model, the variable was analyzed (as a metric variable, as well as a dichotomized variable) in univariate mixed models. The significance level has been set to alpha = 0.05. Due to limited sample size, we did not include interaction terms with device in the model. We assumed that there is a shift in mean values between devices, but that the associations of the independent variables with sensitivity are similar in both devices. Note that the analyses and hence the interpretation of the p-values are exploratory.

## Results

A total of 3.545 sensitivity values from iAMD eyes presenting with pathognomonic anatomical biomarkers were included in the final models. The mean patient age was 76 ± 7 years. Table 1 represents the descriptive statistics of the morphological biomarkers with EZ and ONL thickness and in addition of HRF volume, SDD volume and drusen volume for all stimuli points with values > 0. SDD volume with > 0 measured in 802/3.562 (22.51%) stimuli points, drusen volume > 0 in 1.459/3.545 (41.16%), and HRF volume > 0 in 127/3.545 (3.6%). Mean fixation stability on MAIA (IQR) was 87% (11.4) within the central 2° (P1) and 98% (4.5) within the central 4° (P2). The mean fixation for MP3 was 85.5% (14.9) within the central 2° (P1) and 95% (5.8) within the central 4°.

**Table 1:** Descriptive statistics for all topographic OCT-based biomarkers with mean ± SD for normally distributed values and median [min–max] for skewed data.

	MP-3 Run 1	MP-3 Run 2	MAIA Run 1	MAIA Run 2	Observations
Sensitivity (dB)	26.13 ± 3.42	26.37 ± 3.42	22.70 ± 3.71	22.55 ± 3.67	3.600
EZ thickness (μm)	28.91 ± 7.07	28.80 ± 7.25	28.79 ± 6.90	28.73 ± 6.99	3.545
ONL thickness (μm)	64.79 ± 20.09	64.52 ± 19.93	63.46 ± 19.28	63.43 ± 19.22	3.545
Drusen (nl)*	0.12 [0.00–3.62]	0.12 [0.0–3.50]	0.10 [0.0–3.82]	0.10 [0.0–3.85]	1459
SDD (nl)*	0.27 [0.00–0.87]	0.29 [0.00–0.88]	0.22 [0.00–0.98]	0.22 [0.00–0.82]	802
HRF (nl)*	0.09 [0.06–0.44]	0.09 [0.06–0.41]	0.09 [0.06–0.47]	0.07 [0.06–0.42]	127

*Drusen, SDD and HRF volume for values > 0. SDD = subretinal drusenoid deposit, ONL = outer nuclear layer, EZ = ellipsoid zone.

### Point-wise structure/function correlation

**Fig. 4:**
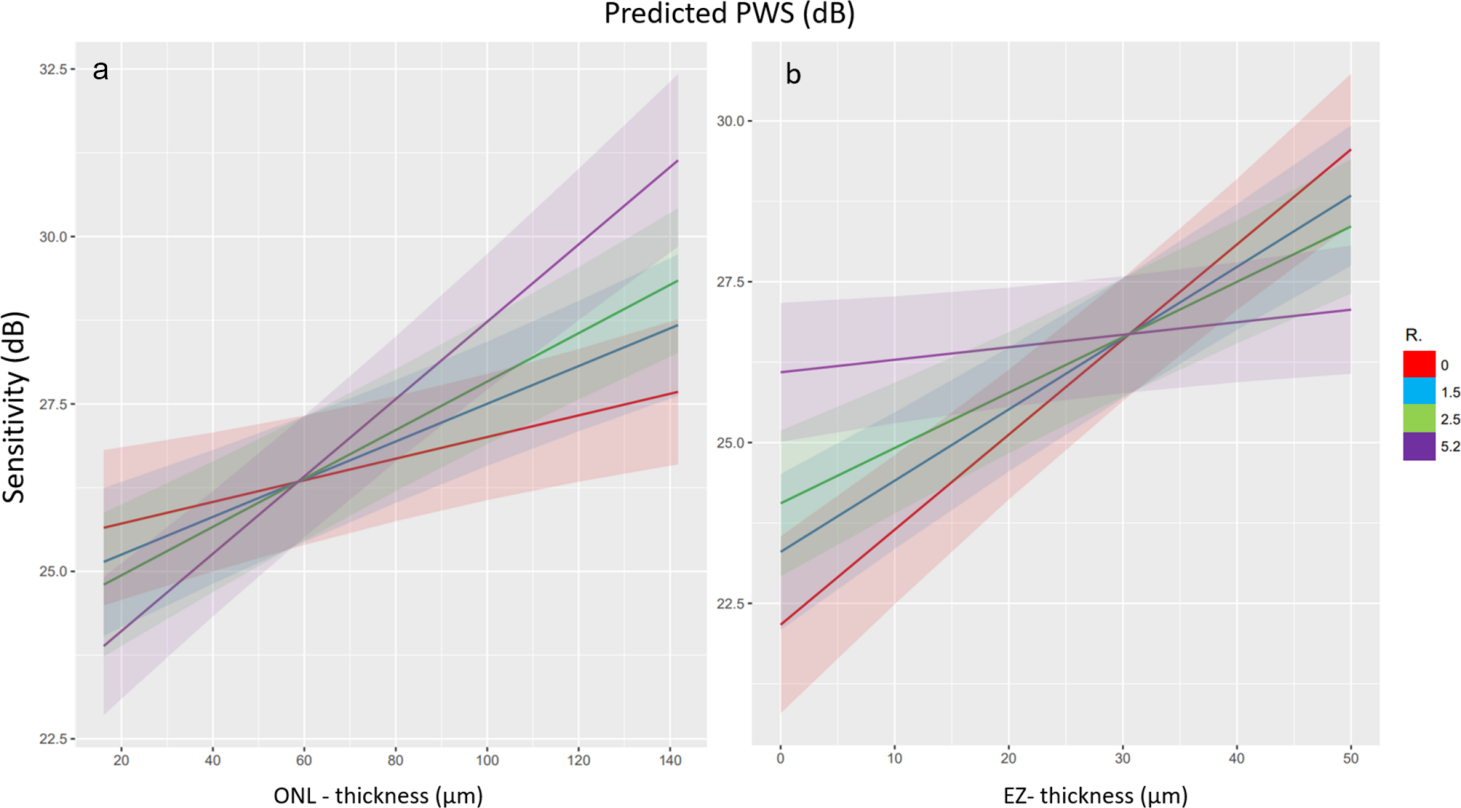
Effect of EZ (**b**) and ONL thinning (**a**) on focal retinal sensitivity dependent on retinal eccentricity (R°). Note that all macular locations showed a consistent correlation, but only the slope at 5.2° eccentricity did not reach significance for the EZ condition, yet there was a strong trend (*p* = *0.055*). However, ONL thickness at 5.2° did reach significance (*p* < 0.001). *EZ* ellipsoid zone, *ONL* outer nuclear layer thickness.

Results of multivariable mixed effect models according to the smallest BIC (*Marginal R*^2^_*GLMM*_ = 0.312) are shown in Table 2. Sensitivity was generally lower when measured with the MAIA compared to the MP-3 device. With increasing drusen and HRF volumes, functional sensitivity decreased. A significant interaction between ONL thickness and eccentricity, as well as between EZ thickness and eccentricity was observed. Both features, ONL thickness and EZ thickness showed a positive association with sensitivity levels at the foveal center point (R° = 0). Note that extreme leverage points were observed in one patient that affected especially the estimate of drusen volume. Characteristics of this patient are in line with the iAMD definition by Ferris et al. and presented in Fig. 2. Figure 4 demonstrates the slope of the EZ thickness and ONL thickness effect on PWS with increasing eccentricity.

**Table 2:** Mixed effect model with impact of topographically quantified neurosensory biomarkers on retinal sensitivity measured with both MP devices.

Variable	Estimate	95% LL	95% UL	*p*
MAIA versus MP3	− 3.553 dB	− 3.730	− 3.376	< 0.001
Drusen volume	− 0.632/nl	− 1.057	− 0.207	< 0.001
HRF volume	− 9.535/nl	− 12.755	− 6.316	< 0.001
ONL thickness (at 0°)	0.016/μm	0.006	0.026	< 0.001
ONL thickness (at 1.5°)	0.028/μm	0.019	0.037	< 0.001
ONL thickness (at 2.5°)	0.036/μm	0.027	0.045	< 0.001
ONL thickness (at 5.2°)	0.058/μm	0.047	0.069	< 0.001
EZ thickness (at 0°)	0.148/μm	0.114	0.181	< 0.001
EZ thickness (at 1.5°)	0.111/μm	0.084	0.138	< 0.001
EZ thickness (at 2.5°)	0.086/μm	0.063	0.109	< 0.001
EZ thickness (at 5.2°)	0.019/μm	− 0.0004	0.039	0.055
ONL: R°	0.008	0.006	0.010	< 0.001
EZ: R°	− 0.025	− 0.031	− 0.019	< 0.001

*ONL* outer nuclear layer, *EZ* ellipsoid zone, *HRF* hyperreflective foci, *MAIA* MP3, 95% LL/ 95% UL lower/ upper limit of the 95% confidence interval.

### Correlation between independent variables

A moderate to high correlation was shown between ONL thickness and eccentricity (*r*_*s*_ = − 0.67). A moderate correlation was observed between SDD volume and EZ thickness (*r*_*s*_ = 0.45) and drusen volume and eccentricity (*r*_*s*_ = − 0.45).

### Impact of subretinal drusenoid deposits

**Fig. 5:**
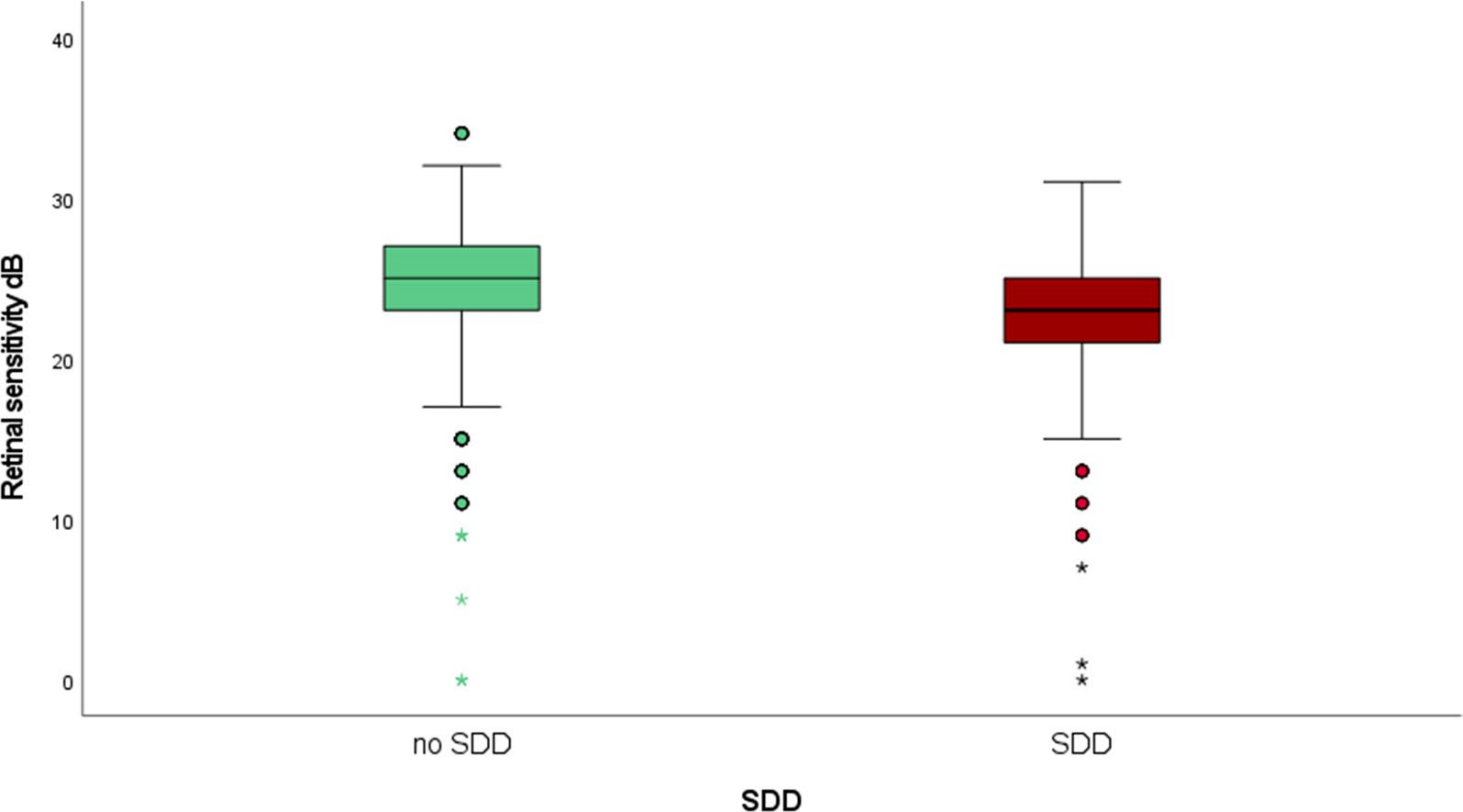
Boxplots of point-wise retinal sensitivity in stimuli points with no SDD (green, left) and SDD (red, right).

Mean PWS was 26 ± 4 dB and 23 ± 4 dB in stimuli without and with SDD, respectively, with a significant difference in retinal sensitivity between stimuli with and without SDD (estimate − 0.71 [− 1.04; − 0.38], *p* < 0.001) as displayed in Fig. 5. An exploratory univariate model showed that SDD volume had a significant negative effect on retinal sensitivity with an estimate of − 1.18db/nl [− 2.10; − 0.26], *p* = 0.012.

## Discussion

Pixelwise co-registration between focal changes on SD-OCT of clinically relevant biomarkers in iAMD showed a significant reduction in retinal sensitivity with neurosensory features such as decreased EZ and ONL thickness as well as increased HRF and drusen volume using two different MP devices, MP-3 and MAIA, respectively. Our proof-of-principle study demonstrates that automated DL-based algorithms are able to localize and quantify subclinical biomarkers on a pixel level with a significant topographic association with retinal function. Moreover, it is the first investigation to include pixel-wise measurements of SDD and multiple high-risk biomarkers quantified by DL on SD-OCT imaging, while also accounting for the effect of age and distance from the fovea (eccentricity in °).

Mean retinal sensitivity (overall RS) was reported to be reduced in eyes with intermediate AMD compared to controls^31^and discussed as a potential prognostic factor of disease progression^5^. Nonetheless, knowledge about localized functional changes in RS in respect to specific biomarkers adds information on disease activity of iAMD for potential structural/functional endpoints in clinical practice and trials^32,33^. Therefore, various groups analyze localized RS changes over quantified high-risk biomarkers of iAMD^34,35^. Expanding on previous concepts, we demonstrated in a systematic manner that retinal function is strongly impacted by focal EZ thickness, accurately defined as space between the ellipsoid zone and the outer border of the interdigitation zone^3,36^. This definition of the EZ encompasses the mitochondria-rich bodies of the photoreceptors, which are morphological predictors of metabolic health and are responsible for the integrity and reflectivity of this outer retinal band on OCT imaging^37,38^. Landa et al. showed a strong correlation between EZ integrity in exudative and non-exudative AMD^11^, while Saßmannshausen et al.^3^ indicated, that an overall thinning of “*PR segments*” significantly influences function under mesopic and scotopic conditions. Compared to former work on structure/function correlation of EZ thickness in intermediate AMD^3^, our MP grid provided the highest density of stimuli points in the foveal area with the highest cone density^39^. Moreover, statistical analyses accurately accounted for the important interaction between EZ thickness and foveal eccentricity (*p* < 0.001). EZ thinning leads to a significant decrease in PWS dependent on eccentricity in both, mesopic and photopic conditions and must be considered during structure/function correlations as well as it confirms the association by differential retinal locations. Notably, previous histological studies demonstrated in a small cohort with non-exudative AMD that cones at the foveal center are more resistant to cell death^39^. However, our results strongly suggest that EZ thinning in this central cone-dominated region significantly reduces retinal function. Concomitantly, pre-existing histological evidence demonstrated cone and rod loss in parafoveal areas at 0.5–1 mm, which corresponds tightly to our observations at 1.5° (approx. 0.4 mm, *p* < 0.001) and 2.5° (approx. 0.7 mm, *p* < 0.001). Our study did not include scotopic testing, which might explain the flattening of the EZ-thickness slope (Fig. 4) and a lower effect of EZ thickness at higher foveal eccentricity with rods predominantly located at 5.2° (approx. 4.5 mm). Finally, the effect of an automatically measured EZ thickness is in full accordance with previous works that utilized qualitative (*present vs. absent*) grading and time-consuming manual layer correction, yet due to its fully automated nature is easily usable in large cohorts^3,11,17^.

The ONL encompasses the photoreceptor cell nuclei and was described as an important biomarker in early and advanced AMD affecting also clinically non-atrophic areas^23,40^. Histological studies described enhanced thinning of the ONL with disease progression to late atrophic AMD^41^. In agreement with current publications under mesopic and scotopic conditions, we demonstrated that topographic ONL thinning is associated with lower PWS in mesopic as well as photopic conditions^3^. Quantification of the ONL condition during long-term follow-up including a potential conversion to late-stage atrophic AMD may be challenging. Histologic analyses suggested that areas with advanced atrophy of neurosensory layers are affected by ELM loss^42,43^. As the ONL appears hyperreflective on OCT imaging, complete ONL integrity loss may be challenging to define on OCT when the ELM is absent and parts of the hyperreflective ONL remain present^41^.

The impact of drusen on retinal sensitivity is controversially discussed in the current literature and remains ambiguous^16,17^. Earlier work based on qualitative drusen grading (*present vs. absent*) did not reveal a significant decrease in retinal function in drusen areas under mesopic conditions^17^. Pixel-wise measurements of drusen volume or the “*RPE-Drusen-complex*” showed a significant decrease in point-wise sensitivity over drusen using different MP devices under mesopic and scotopic conditions^3,16,44^. Our findings suggest that there is a significant negative effect of increasing drusen volume (− 0.632/nl [− 1.057–0.207], *p* < 0.001). Thorough analyses of the data revealed one patient with a point-wise drusen volume > 3 nl. This patient presented with larger drusen in the central 1 mm compared to the respective cohort, but was in line with the iAMD definition by Ferris et al. ^19^(example in Fig. 2). This outlier highlights the interindividual discrepancies in iAMD morphology and the need for a reliable quantification of progression-relevant biomarkers to assess disease activity in a personalized manner, not only in late-stage disease, but already in early disease stages. Also, analyzing the association between PWS and the metric variable drusen volume, a statistically significant but small effect of drusen volume was observed (− 0.6 dB/nl). The clinical and biological meaning of such subclinical PWS changes is not yet fully understood. Advances in deep-learning are the key to unlocking pathophysiological information from OCT volumes and correlating each biomarker to topographic function to objectively establish the role of a vast spectrum of high-risk biomarkers for both, function and progression. Also, it is of utmost importance to adjust for the presence of coexistent biomarkers as presented in this study. To expand on our results, larger cohorts with longitudinal data are necessary to truly establish the clinical relevance of e.g. drusen and understand their role for functional impairment, which obviously requires automated tools. Especially drusen growth and regression and its impact on EZ thickness and integrity needs to be quantified in longitudinal data.

Although the HRF effect appears large (− 9.54 [− 12.75; − 6.32], *p* < 0.001), the median HRF volume per stimulus was 0.07–0.09 nl (Table 1). Therefore, interpretation of the threshold as changes with 0.1 nl/dB is more suited and adjusted HRF estimates would be − 0.95/0.1 nl [− 1.28; − 0.63]. Notably, HRF mostly overly areas of RPE disruption and are generally considered to correspond to RPE migration, an important quantifiable SD-OCT biomarker for progression to late-stage disease^45–47^. We hypothesize therefore that the effect of HRF on PWS includes the effect of RPE irregularities/loss as well as concomitant EZ integrity loss as seen in Fig. 2. Obviously, such ultrastructural changes can only be investigated using highly precise algorithms. A high amount of value 0 for HRF and drusen volume might lead to instable estimates in the final model. We added a model with analysis of drusen and HRF presence to the supplement (Supplementary Table 1). The significance level of all biomarkers remained equal, only drusen presence did not have any significant effect compared to the quantitative model in Table 2.

**Fig. 6:**
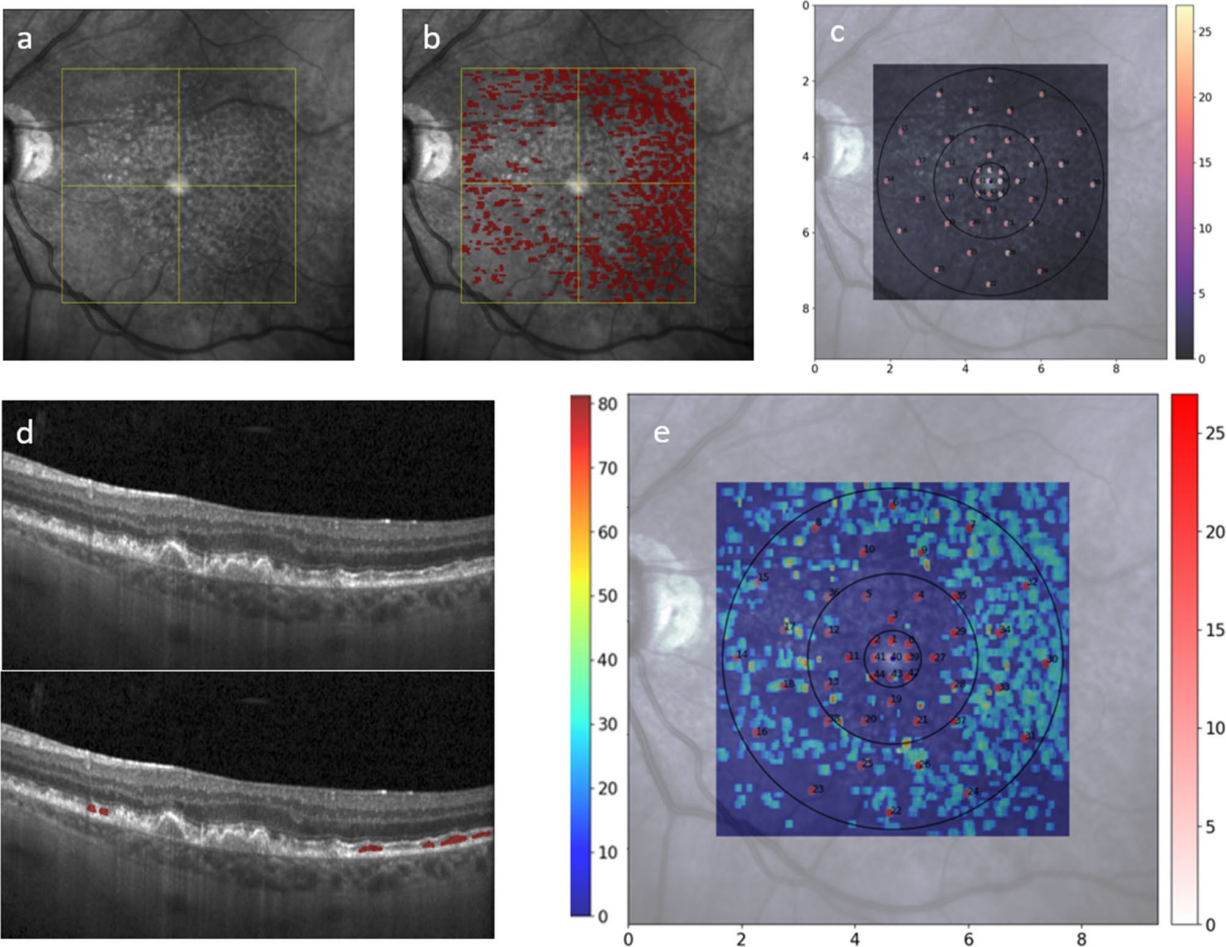
Example of patient with SDD visible in NIR without manual segmentation (**a**) and with manual segmentation (**b**) and the respective B-scan of the foveal centerpoint (**d**). The position of the MP grid is represented in (**c**) and the combination of the SDD segmentation heatmap and the position of the MP grid is displayed in (**e**). The numbers on the MP grid represent the numbers of the MP stimuli, not the sensitivity values. *SDD* subretinal drusenoid deposit, *MP* microperimetry, *NIR* near infrared reflectance.

Previous analyses of the SDD area on NIR showed a significant negative effect of the overall amount of the SDD area on retinal sensitivity^15,48^. Kumar et al. demonstrated that SDD presence and extent led to significantly lower SDD values throughout the entire OCT volume and postulated an enhanced degeneration in eyes with SDD, while the correlation between PWS and SDD remained unclear^15^. Steinberg et al. compared PWS in stimuli over SDD on NIR with healthy areas and found a significant difference between the respective areas^14^. Our findings are not only of high precision, but also pivotal in regard to understanding SDD, as we were able to quantify SDD in OCT B-scans, not only NIR (Fig. 6). In this study, SDD volume was calculated based on thorough manual annotation on every single SDD in study eyes and over all 97 B-scans per OCT volume equivalent to 1.164 B-scans. A point-wise decrease in retinal sensitivity over SDD reached statistical significance in an exploratory univariate model. However, SDD was not included in the final model, due to its potential multicollinearity with EZ thickness. Notably, SDD were present in only 22% of the analyzed stimuli points, which might weaken their effect. Moreover, the EZ thickness measurement encompasses parts of the SDD material in B-scan areas with SDD present. Consequently, the negative effect of SDD on retinal function might also be included in the EZ thickness measurement statistics. Examinations using advanced EZ algorithms in larger cohorts will allow to fully understand the change in PWS over SDD and their impact on EZ health^49^.

We included all measured sensitivity values from both devices, MP-3 and MAIA. Both tools were used using their standard settings, with photopic conditions on MP-3 and mesopic on MAIA with slightly deviating decibel range, 0–34 dB (MP-3) versus 0–36 dB (MAIA), respectively. Firstly, differences in the decibel range and different background luminosities are the main reason for the significant difference of − 3 dB between the two devices (*p* < 0.001). Secondly, lower sensitivity values in MAIA could be explained by the hypothesis that rod degeneration occurs before cone degeneration in intermediate AMD^39^. Although the decibel range is dependent on the background luminosity, rod-mediated signals in mesopic test conditions may be greatly impacted by these early changes and lead to lower sensitivity values. This observation is of high clinical relevance for the design of future clinical trials and functional endpoint analyses.

The strengths of our analyses included DL-supported measurements of multiple progression-relevant biomarkers of intermediate AMD and standardized PWS measurements throughout more than 3.500 B-scans for two MP devices. In addition, our study was carried out in a prospective manner with a novel approach of co-registration between PWS and OCT volumes using advanced image registration algorithms. Hence, we provide PWS value changes over several high-risk biomarkers, while accounting for physiological co-founders such as age and eccentricity. The limitation is our small cohort size and the lack of longitudinal data. Longitudinal studies are necessary to further establish the quantitative impact of these subclinical biomarkers on progression and function during iAMD progression and conversion. Larger trials are ongoing and will provide extensive data using the procedures developed and described in this exploratory analysis^49^. Manual ONL corrections and SDD annotations were performed with qualified human expertise, but a subjective aspect is inevitable in difficult cases. Also, another limitation of general use of MP in early and intermediate AMD remains that subclinical changes might not be easily assessed within the range of the MP system, as most values remain within the upper range for both, especially in mesopic and photopic testing^3,50^. However, statistically significant localized changes of PWS in our cohort add to preexisting evidence on associations between structural OCT changes and MP^3,16,35,51^Devices used in our study are free of previously described ceiling effects in older instruments^52^. A precise co-registration between MP and OCT was performed at an expert level with certified human expertise. However, minimal deviations in the analyzed area are inevitable due to the device-specific follow-up and eye-tracking software, the limited B-scan density (stimuli might be located between two adjacent B-scans), and the localization of prominent anatomical loci.

In conclusion, this proof-of-principle study contributes important evidence on the functional impact of early retinal sensitivity decline in areas with EZ and ONL thinning, as well as over HRF and drusen in a topographical and quantified manner in the ongoing path of identifying the effects of subclinical biomarkers in iAMD. Advanced automated DL-based tools will offer pivotal insights for study endpoints to intervene in an early and timely way and identify the relevant therapeutic targets.

## Supplementary Information

Supplementary Figure.

Supplementary Table 1.

## Data Availability

Data will be available from the corresponding author upon resealable request.
