# Supplementary figures and images for: Topographic and quantitative correlation of structure and function using deep learning in subclinical biomarkers of intermediate age-related macular degeneration
Source: Sci Rep. 2024 Nov 15;14:28165. doi: 10.1038/s41598-024-72522-9 (PMC11568137; doi:10.1038/s41598-024-72522-9)

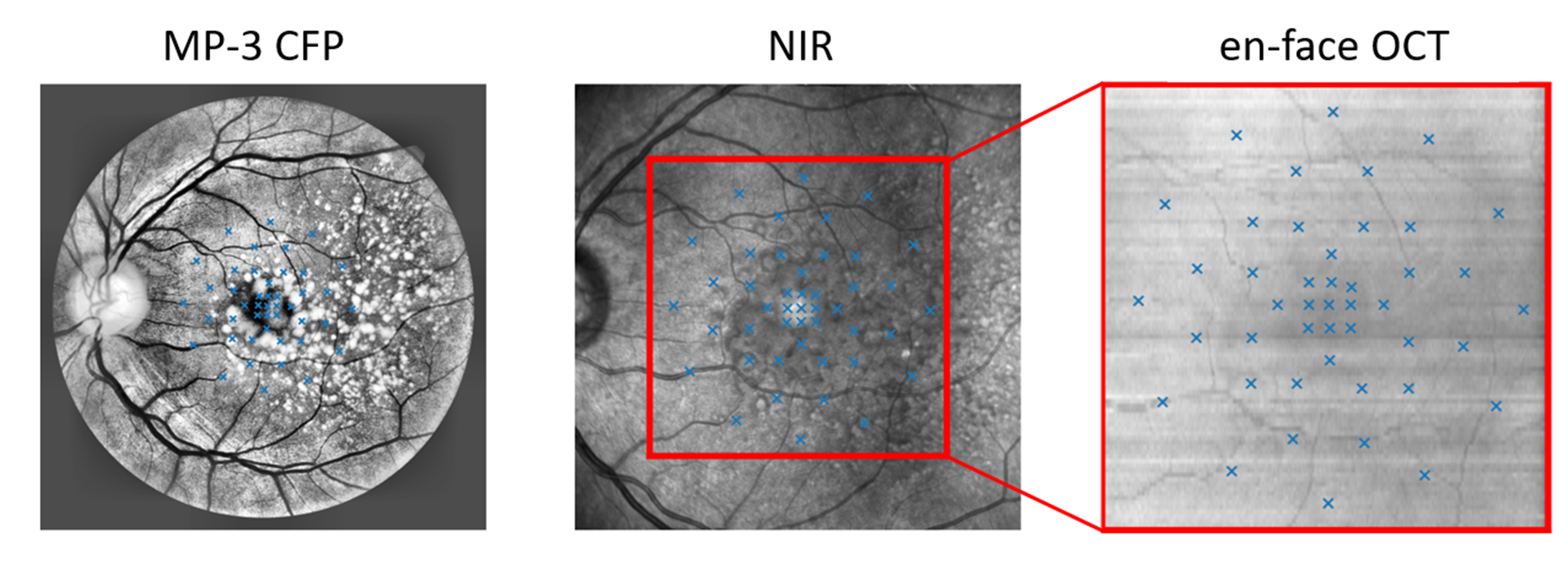

Supplement: Supplementary file 1 — Supplementary Figure. [file 41598_2024_72522_MOESM1_ESM.png]
