# Supplementary material for: Topographic and quantitative correlation of structure and function using deep learning in subclinical biomarkers of intermediate age-related macular degeneration
Source: Sci Rep. 2024 Nov 15;14:28165. doi: 10.1038/s41598-024-72522-9 (PMC11568137; doi:10.1038/s41598-024-72522-9)
Supplement: Supplementary file 2 — Supplementary Table 1. [file 41598_2024_72522_MOESM2_ESM.docx]

**Supplementary material:**

| **Variable** | **Estimate** | **95% LL** | **95% UL** | **p** |
| --- | --- | --- | --- | --- |
| MAIA vs. MP3 | -3.55 | -3.73 | -3.37 | < 0.001 |
| HRF volume (ref = 0) | -1.009 | -1.56 | -0.45 | < 0.001 |
| ONL – thickness (5.2°) * | 0.065 | 0.054 | 0.075 | < 0.001 |
| EZ – thickness (5.2°) * | 0.036 | 0.017 | 0.054 | < 0.001 |
| R° * | 0.098 | 0.041 | 0.16 | < 0.001 |
| ONL : R° | 0.008 | 0.006 | 0.010 | < 0.001 |
| PR : R° | -0.031 | -0.036 | -0.025 | < 0.001 |

**Supplementary Table 1:** Multivariable mixed model with impact of deep-learning quantified biomarkers with HRF and drusen as dichotomic variables (reference 0) measured with two MP devices. Drusen presence has been eliminated from the final model to improve model performance based on BIC. ONL = outer nuclear layer, EZ = ellipsoid zone, HRF = hyperreflective foci, R = retinal eccentricity (°), *Effect of EZ and ONL-thickness at R. ° = 5.2, effect of retinal eccentricity at ONL = 64 μm and PR = 28.81 μm, 95% LL/ 95% UL = lower/ upper 95% confidence limit
